# Influence of a *phyA* Mutation on Polyamine Metabolism in *Arabidopsis* Depends on Light Spectral Conditions

**DOI:** 10.3390/plants12081689

**Published:** 2023-04-18

**Authors:** Altafur Rahman, Judit Tajti, Imre Majláth, Tibor Janda, Sylva Prerostova, Mohamed Ahres, Magda Pál

**Affiliations:** 1 Agricultural Institute, Centre for Agricultural Research, Eötvös Loránd Research Network, 2462 Martonvásár, Hungary; 2 Laboratory of Hormonal Regulations in Plants, Institute of Experimental Botany, Czech Academy of Sciences, 11720 Prague, Czech Republic

**Keywords:** blue light, phytochrome, far-red light, spermine

## Abstract

The aim of the study was to reveal the influence of *phyA* mutations on polyamine metabolism in *Arabidopsis* under different spectral compositions. Polyamine metabolism was also provoked with exogenous spermine. The polyamine metabolism-related gene expression of the wild type and *phyA* plants responded similarly under white and far-red light conditions but not at blue light. Blue light influences rather the synthesis side, while far red had more pronounced effects on the catabolism and back-conversion of the polyamines. The observed changes under elevated far-red light were less dependent on PhyA than the blue light responses. The polyamine contents were similar under all light conditions in the two genotypes without spermine application, suggesting that a stable polyamine pool is important for normal plant growth conditions even under different spectral conditions. However, after spermine treatment, the blue regime had more similar effects on synthesis/catabolism and back-conversion to the white light than the far-red light conditions. The additive effects of differences observed on the synthesis, back-conversion and catabolism side of metabolism may be responsible for the similar putrescine content pattern under all light conditions, even in the presence of an excess of spermine. Our results demonstrated that both light spectrum and *phyA* mutation influence polyamine metabolism.

## 1. Introduction

Light, as the major energy source for plants, is one of the most important environmental factors, and its characteristics, namely the intensity, the spectral composition, and even the direction affect plant growth and development, including net photosynthesis and chemical composition. These changes, in turn, modify plant morphology and tissue anatomy and, in the long term, influence plant biomass parameters and reproduction-related rates [1]. Plants sense different wavelengths using distinct photoreceptors; among them, one superfamily is phytochromes (Phys), the receptors of red/far-red light [2]. Angiosperm Phys are divided into two groups; type I is characterized as light labile, while the members of type II are light stable [3]. In *Arabidopsis*, there are five Phy genes encoding the apoproteins of Phy A-E [4]. The PhyA protein is considered primarily as a far-red sensor, while the PhyB is rather a red sensor [5]. The effects of *phyA* mutation have been studied with the usage of, for example, T-DNA insertion *Arabidopsis* mutants. Besides the effect of far-red light and darkness in mutant plants on dormancy and germination or hypocotyl elongation [6,7,8], the role of *phyA* mutation has also been investigated in jasmonic acid-mediated defense response against *Botrytis cinerea* [9], in peroxisome proliferation during photomorphogenesis [10], and in cold acclimation under different light quality and intensity conditions in *Arabidopsis* [11].

PhyA-regulated responses involve metabolite and gene expression fine-tuning mechanisms [12,13,14,15]. Investigation on *phyA* and *phyB* mutant *Arabidopsis* plants also revealed that PhyA and PhyB signaling has essential roles in the control of primary metabolism, including starch and sugar content, and other metabolites involved amino acids, polyamines (PAs) and the members of the tricarboxylic cycle in response to light [14], and phytohormone levels [11]. The most detailed study on the relationship between *phyA* mutation and PA metabolism performed under white and far-red light conditions revealed the involvement of PhyA in putrescine (PUT) biosynthesis and its role in the regulation of S-adenosylmethionine decarboxylase 2 and 4 in response to far-red light [12]. Some interesting results also revealed the role of PhyA in mediating the blue light/UV-A photoresponses during chloroplast biogenesis [16] and phototropism [17]. Mesophyll-specific PhyA is involved in the blue-light-dependent regulation of anthocyanin levels [18]. Investigation on quintuple *phy* mutant (*phyA phyB phyC phyD phyE*) *Arabidopsis* plants also revealed that Phys play a role in blue-light-mediated stem elongation and the associated shade-avoidance responses [19] or chloroplast development in *Arabidopsis* under blue light [20]. However, the involvement of Phys in blue light-induced processes is still less known.

PAs are small N-containing polycationic compounds with various roles in plant physiological and molecular processes, such as biosynthesis, structural maintenance, stabilization and function of proteins and nucleic acids, photosynthesis, plant growth and development, and stress signaling [21]. Thus, the maintenance of a proper level of PA pool is important for normal plant growth and development [22]. In plants, the diamine PUT can be synthesized mainly by ornithine decarboxylase (ODC) or arginine decarboxylase (ADC), with one well-known exception, as in *Arabidopsis* the ODC pathway is lacking, but this species displays two ADCs genes (*AtADC1* and *AtADC2*) [23]. Light and several stress factors have been shown to have more influence on *ADC2* promoter activity [24,25]. PUT can be converted to the triamine spermidine (SPD), which can be further converted to the tetramine spermine (SPM) by the repetitive addition of an aminopropyl moiety in reactions catalyzed by two closely related but specific enzymes, SPD synthase (SPDS) and SPM synthase (SPMS) [22]. The *Arabidopsis* genome carries two genes encoding SPDSs (*AtSPDS1* and *AtSPDS2*) and one encoding SPMS (*AtSPMS*) [26]. The expression of *AtSPDS1* and *AtSPDS2* have been reported to be constitutive in all organs [27], but *AtSPDS2* was found to be inducible by plant hormones, such as indoleacetic acid, gibberellin A3 and abscisic acid [28]. The fine-tuning of the PA metabolism is achieved by the balance between the synthesis and catabolism, uptake and conjugation of PAs, and it can also be assumed that light may affect not only the synthesis but also the catabolism of Pas [24]. Among the ten annotated Copper-Containing Amine Oxidase (*CuAOs)* genes in *Arabidopsis*, *AtCuAO1*, *AtCuAO2* and *AtCuAO3* have been well characterized. These genes encode functional CuAOs that use PUT and SPD as substrates and are responsible for terminal catabolism. CuAO1 has extracellular, while CuAO2 and CuAO3 have peroxisomal localization. The three genes present different expression profiles [29]. *AtCuAO1* transcripts were abundant in rosette leaves, and the expression increased continuously during development. Similarly, the expression pattern of *AtCuAO3* increased during development, and its transcript level was high both in the leaves and in the stems. While the expression level of *AtCuAO2* was abundant rather in stems and was low in the leaves, in addition did not increase during the developmental stages. Plant hormone treatments, such as abscisic acid or salicylic acid, also induced the gene expression of *AtCuAO1* and *AtCuAO3* but not that of *AtCuAO2* [29]. In *Arabidopsis*, all the known polyamine oxidases (PAOs) are responsible for the back conversion of higher PAs. Characterization of five PAO isoforms in *Arabidopsis* revealed that *AtPAO1* has a specific role in flowers, *AtPAO2* was expressed in shoot and root meristems, and to a greater extent at later growth stage in the rosette, stem and in flowers. The expression of *AtPAO3* was constitutive in the whole plant body but was the highest in flowers. *AtPAO4* was expressed in young and adult plants, especially in the roots and floral organs, while *AtPAO5* expression was observed in all the organs throughout various growth stages [30]. *AtPAO1* and *AtPAO5* are located in the cytoplasm, while *AtPAO2*, *AtPAO3*, and *AtPAO4* are in peroxisomes. *AtPAO1* and *AtPAO5* prefer thermospermine, whereas *AtPAO4* has higher substrate specificity for SPM and converts it to SPD, while *AtPAO2* and *AtPAO3* mainly convert SPD to PUT [31,32]. *AtPAO2* and *AtPAO5* have been reported to be inducible by, for example, salt stress and plant hormones or Pas [33,34,35,36].

It has also been demonstrated that the actual PA pool is influenced by light intensity and light composition [37,38,39]. In addition, it was found that far-red and blue lights induced opposite responses in PA metabolism-related gene expression in wheat plants [39]. Pas increasingly accumulated in tomato leaves from the beginning of illumination [40]. Furthermore, light-responsive elements in the promoter region of genes involved in PA synthesis, such as *SAMDC* encoding S-adenosylmethionine decarboxylase, have been identified in *Arabidopsis* [41].

Although there are some limited results on the changes in PA metabolism in *phy* mutant *Arabidopsis* plants, these studies focused on the modifying effect of the red/far-red ratio with the investigation of only dedicated parts of PA metabolism (just one polyamine compound, or only one of the synthesis/catabolite gene) (reviewed in [24]). Furthermore, these studies examined the effects of the mutation during alternating light conditions or pulse-like light treatments. However, the relationship between light perception and PA metabolism has still not been fully understood.

The present study aimed to reveal the influence of PhyA on PA synthesis and catabolism. Therefore, PA metabolism changes in *phyA* insertion mutant *Arabidopsis* plants were compared to the wild type. As PhyA responds to far-red, plants were exposed to white light, as well as white light with an enhanced far-red spectrum. Moreover, the effect of supplemental blue light also was investigated because some studies suggested that Phys play a role in special blue-light-mediated responses [16,17,19]. In order to estimate the direct impact of PhyA on PA catabolism, some plants were supplemented by exogenous SPM.

## 2. Results

### 2.1. Measurements of the Shoot Weight and Chlorophyll-A Fluorescence Induction Parameters

**Figure 1:**
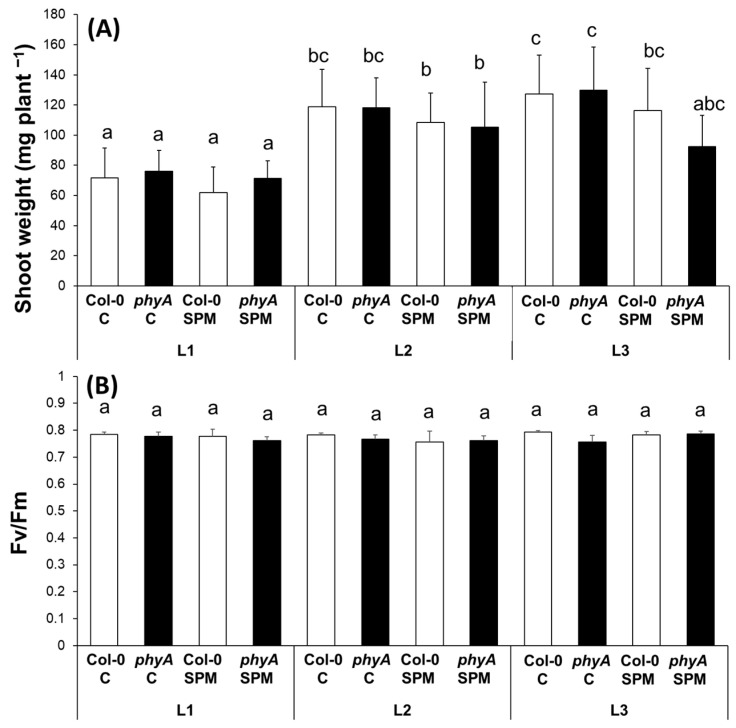
Shoot weight (**A**) and the maximum quantum efficiency of photosystem II (Fv/Fm) chlorophyll-a fluorescence induction parameter determined at the steady state level of photosynthesis of the leaves (**B**) of wild type (Col-0) and mutant (*phyA*) *Arabidopsis* plants grown under three different light regimes (L1: blue%: 19.139, green%: 30.62, red%: 48.8 and far-red%: 1.44; L2: blue%: 19.57, green%: 29.57, red%: 40.43 and far-red%: 10.43; L3: blue%: 39.38, green%: 28.76, red%: 30.53 and far-red%: 1.33) treated with or without 0.5 mM spermine (control: C and spermine: SPM). Values are means ± SD (n = 14 for shoot weight and n = 5 for Fv/Fm). Different letters indicate statistically significant differences at *p* < 0.05 level, using Duncan’s post hoc test.

Wild type Col-0 and *phyA* mutant Arabidopsis plants were grown at different light spectral conditions (the light source for L1: “white light” was a continuous wide-spectrum LED, in the case of the L2 regimen: “red light”, elevated far-red component was applied, while under L3 light condition: “blue light” the blue light component was the most dominant), with or without exogenous SPM treatment. In order to characterize the physiological status of the plants, shoot weight and the maximum quantum efficiency (Fv/Fm) chlorophyll-*a* fluorescence induction parameter representing the maximum quantum efficiency of Photosystem II (PSII) was measured first. The *phyA* mutation did not significantly influence the shoot weight under any of the light conditions (Figure 1A). However, the L2 and L3 light treatments increased it in both genotypes compared to the control, white light (L1), indicating that light quality has more influence on plant growth rate than the mutation. The one-day SPM treatment also did not significantly influence the shoot weight of the plants under different light conditions (Figure 1A). The Fv/Fm parameter was around 0.8, and neither the treatments nor the genotypes influenced it, indicating that the plants were under optimal growth conditions (Figure 1B).

Besides the Fv/Fm, the actual quantum yield [Y(II)] of PSII was also measured. Under L1 and L2 light conditions, no differences were detected between the wild type and the mutant plants in Y(II). Under L3 light, slightly lower Y(II) was measured for the *phyA* mutants compared to the Col-0 (Supplementary Figure S1b). It has also been shown that the actual quantum yield was slightly more sensitive to the SPM treatment in the wild genotypes than in the *phyA* mutant plants under L1 and L3 light conditions (Supplementary Figure S1a,b). At L2 treatment, SPM application slightly increased the Y(II) in the *phyA* mutant plants. Despite a few statistically significant differences, these changes were not substantial.

### 2.2. Modulation of Polyamine Content and Polyamine Metabolism-Related Gene Expression by Light Spectra

**Figure 2:**
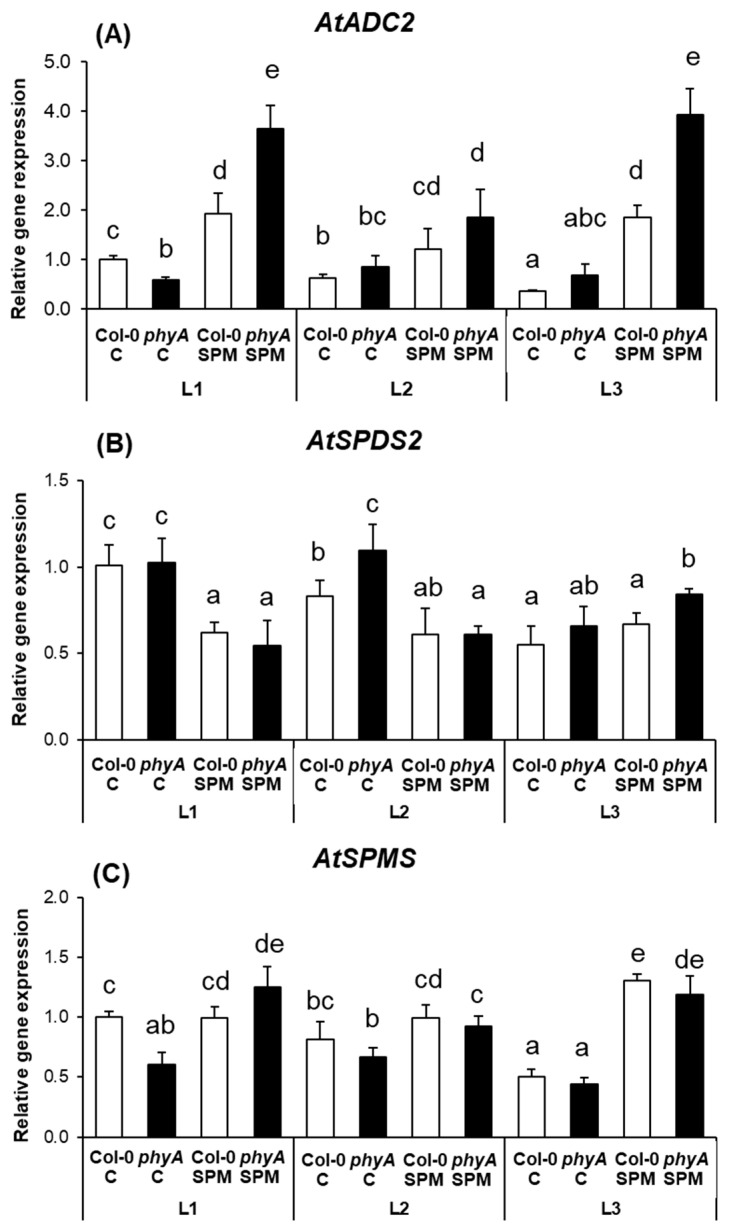
Changes in the expression levels of polyamine synthesis-related genes, namely arginine decarboxylase (*AtADC2*) (**A**), spermidine synthase (*AtSPD2*) (**B**) and spermine synthase (*AtSPMS*) (**C**) of wild type (Col-0) and mutant (*phyA*) Arabidopsis plants grown under three different light regimes (L1: blue%: 19.139, green%: 30.62, red%: 48.8 and far-red%: 1.44; L2: blue%: 19.57, green%: 29.57, red%: 40.43 and far-red%: 10.43; L3: blue%: 39.38, green%: 28.76, red%: 30.53 and far-red%: 1.33) treated with or without 0.5 mM spermine (control: C and spermine: SPM). Values are means ± SD (n = 3). Different letters indicate statistically significant differences at *p* < 0.05 level, using Duncan’s post hoc test.

As light and several stress factors have been shown to be important regulators of *ADC2* promoter activity, here, the *ADC2* gene was in focus. In accordance, we detected downregulated expression of *AtADC2* by L2 and L3 treatments in Col-0 plants (Figure 2A). In addition, the expression of *AtADC2* was lower in the mutant plants under L1 but the same under L2 and L3 light conditions. A much stronger effect was found in the case of SPM-treated plants (Figure 2A). After the SPM application, *AtADC2* expression increased in both genotypes, which suggested that the SPM treatment induced de novo synthesis of PUT. *AtADC2* expression increased, especially in the case of the mutant plants, with a higher degree under L1 and L3 light conditions (Figure 2A).

As the expression of *AtSPDS2* was found to be inducible by plant hormones, such as indoleacetic acid, gibberellin A3 and abscisic acid, here, we focused only on *AtSPDS2*. Without SPM treatment, the *AtSPDS2* transcription levels did not differ between the two genotypes under L1 and L3 conditions, and under L2, it was only slightly higher in *phyA* mutant than in Col-0 (Figure 2B). The lowest *AtSPDS2* expression level was found in both genotypes under the L3 light treatment. SPM treatment decreased the expression of *AtSPDS2* in both genotypes under L1 and L2 conditions, while under L3 conditions, where the expression level was initially lower, no pronounced differences were induced (Figure 2B).

The expression level of *AtSPMS* downregulated in *phyA* mutant compared to the wild type under L1 and L2 light treatments, but not under L3 light conditions, where the expression level was similar to the wild type and initially lower (Figure 2C). SPM treatment induced the highest increment under L3 in Col-0, a slight increase in both genotypes was induced under L2, while under L1, the transcript level of *AtSPMS* increased only in the mutant.

**Figure 3:**
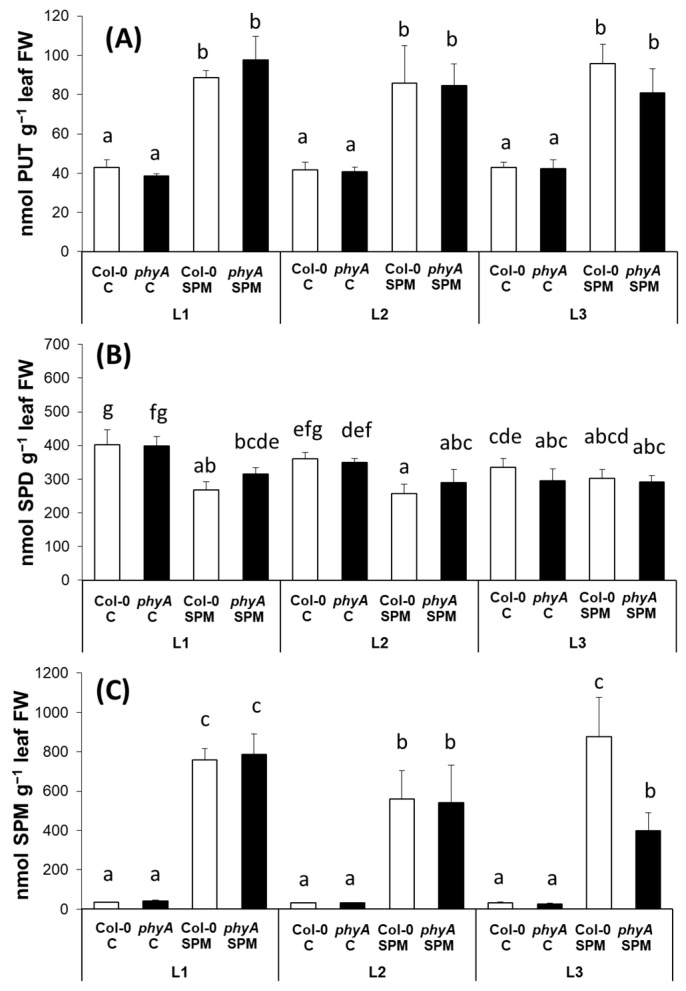
Polyamine contents, namely putrescine (PUT) (**A**), spermidine (SPD) (**B**) and spermine (SPM) (**C**) of wild type (Col-0) and mutant (*phyA*) *Arabidopsis* plants grown under three different light regimes (L1: blue%: 19.139, green%: 30.62, red%: 48.8 and far-red%: 1.44; L2: blue%: 19.57, green%: 29.57, red%: 40.43 and far-red%: 10.43; L3: blue%: 39.38, green%: 28.76, red%: 30.53 and far-red%: 1.33) treated with or without 0.5 mM spermine (control: C and spermine: SPM). Values are means ± SD (n = 3). Different letters indicate statistically significant differences at *p* < 0.05 level, using Duncan’s post hoc test.

PUT content was similar in the wild type and the *phyA* mutant plants under all the light spectral conditions tested here (Figure 3A). A substantial increase was detected in the SPM-treated plants compared to their corresponding control. Despite the well-recognized differences between the expression of *AtADC2* in the two SPM-treated genotypes, as well as under different light regimes, SPM treatment increased the PUT level similarly in both genotypes, which was independent of the light conditions.

Under the present conditions, the most abundant PA in the leaves of *Arabidopsis* plants was SPD (Figure 3B), the amounts of PUT and SPM were about an order of magnitude lower compared to SPD, and no remarkable changes were observed in the SPD levels after the treatments compared to changes in PUT and SPM. In addition, SPM treatment only slightly decreased SPD levels, especially under white and far-red light conditions.

Despite the modifications of the enzyme expression, the pattern of SPM changes did not follow the tendency of *AtSPMS* expression levels. The SPM concentration was similar in both genotypes under the three applied light treatments (Figure 3C), and exogenous SPM increased equally in Col-0 and *phyA* under L1 and L2 light conditions, with a lower extent in the case of L2. Interestingly, under the L3 condition, the SPM accumulation in SPM-treated Col-0 was double that of the *phyA* mutant.

Based on the localization of the CuAO and PAO enzymes, organ-specific expression and plant hormone inducibility of their genes here, the changes in the transcript level of *AtCuAO1* and *AtCuAO3*, in addition to *AtPAO2* and *AtPAO5* were studied.

**Figure 4:**
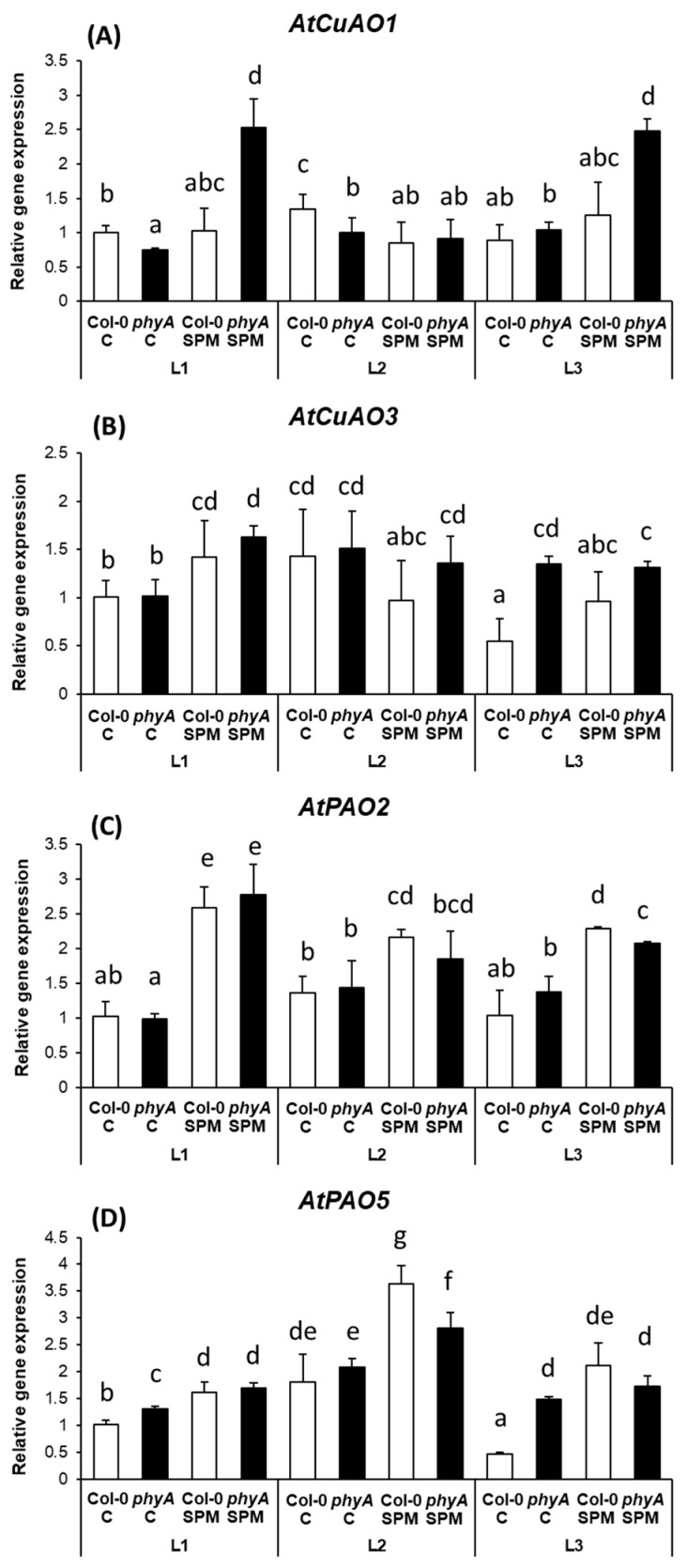
Changes in the expression levels of polyamine metabolism-related genes, namely copper-containing amine oxidases (*AtCuAO1*: (**A**) and *AtCuAO3*: (**B**)), and polyamine oxidases (*AtPAO2*: (**C**) and *AtPAO5*: (**D**)) in the leaves of wild type (Col-0) and mutant (*phyA*) Arabidopsis plants grown under three different light regimes (L1: blue%: 19.139, green%: 30.62, red%: 48.8 and far-red%: 1.44; L2: blue%: 19.57, green%: 29.57, red%: 40.43 and far-red%: 10.43; L3: blue%: 39.38, green%: 28.76, red%: 30.53 and far-red%: 1.33) treated with or without 0.5 mM spermine (control: C and spermine: SPM). Values are means ± SD (n = 3). Different letters indicate statistically significant differences at *p* < 0.05 level, using Duncan’s post hoc test.

Under the present conditions, it was found that the *AtCuAO1* expression was independent of the light condition or the *phyA* mutation. However, in the presence of an excess SPM, *AtCuAO1* expression was induced in the mutant genotypes under L1 and L3 light conditions (Figure 4A).

The expression level of *AtCuAO3* did not show pronounced changes, except downregulation under L3 conditions in Col-0 (Figure 4B). The initially higher *AtCuAO3* expression in *phyA* mutant under the L3 regime might reflect the involvement of PhyA in this process, which is similar to the above-mentioned modifications in *AtCuAO1* expression under SPM treatment combined with L3 conditions. A slight increase of *AtCuAO3* expression was detected after SPM application in both genotypes under L1 light and in Col-0 under L3 (Figure 4B), which also indicates that this process is dependent on the actual level of PAs.

In the present study, the increased SPM content in the leaves, due to the excess of SPM in the nutrition solution, induced the expression of *AtPAO2* under all the light conditions regardless of the genotypes (Figure 4C). Compared to this, the *AtPAO5* levels showed more differences. Its transcript level was initially the lowest under L3 conditions in Col-0 plants (Figure 4D), and its expression was mostly elevated after SPM treatment, especially under L2 conditions in both genotypes, while under L3 conditions only in Col-0 plants. The transcript level of *AtPAO5* was significantly higher in the mutant plant compared to the wild type under L3 conditions, and excess SPM could not increase further it. It should also be noticed that under the L2 condition in both genotypes, the initial level of *AtPAO5* expression was higher than that was detected under L1 conditions in Col-0 or *phyA* mutant plants.

### 2.3. Correlation Analyses and Principal Components Analysis (PCA) of the Measured Parameters

The correlation analyses (Table 1) showed that PUT content was in a close, positive relationship with the SPM content, and both PUT and SPM contents were in a close, positive relationship with the *ADC2*, *SPMS* and *PAO2* expression levels. Additionally, high and positive correlations were detected between the expression levels of *ADC2*, *SPMS*, *CuAO1* and *PAO2*. However, close, negative relations were found between PUT or SPM levels and SPD content, and *SPDS2* transcript level. Furthermore, SPD content also showed a strong, negative correlation with *PAO2* and *PAO5* expression levels.

**Figure 5:**
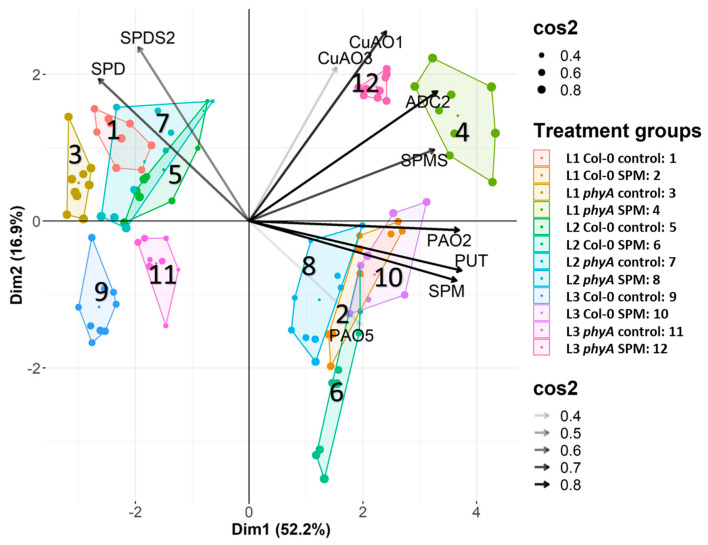
The biplot illustration of PCA analysis was carried out on the standardized concentration values of polyamines and the relative expression values of polyamine metabolism-related genes. Squared cosine (cos2) shows how accurate the representation of our variables or individuals on the PC plane is. Dim1 and Dim2 are equivalent to principal components 1 and 2 (PC1 and PC2). Investigated parameters: PUT: putrescine content, SPD: spermidine content, SPM: spermine content, *ADC2*: expression level of arginine decarboxylase 2, *SPDS2*: expression level of spermidine synthase 2, *SPMS*: expression level of spermine synthase, *CuAO1* and *CuAO3*: expression level of cooper amine-oxidase 1 and 3, *PAO2* and *PAO5*: polyamine oxidase 2 and 5 genes. (Col-0 and *phyA Arabidopsis* plants grown under L1: blue%: 19.139, green%: 30.62, red%: 48.8 and far-red%: 1.44; L2: blue%: 19.57, green%: 29.57, red%: 40.43 and far-red%: 10.43; L3: blue%: 39.38, green%: 28.76, red%: 30.53 and far-red%: 1.33) treated with or without 0.5 mM spermine (control: C and spermine: SPM). Different colors and numbers indicate the 12 treatment combinations.

The principal component analysis (PCA) analysis of the PA metabolism-related parameters (Figure 5) showed great separation in some of the treatment groups based on their measured genetic and biochemical variables. This multivariate analysis reflected that L3 light conditions had a specific effect on PA metabolism. On the other hand, SPM treatment was a more significant factor in the distinction of treated plants. Three variable groups, SPD—*SPDS2*, *ADC2*—*CuAO1*—*SPMS*, and *PAO2*—PUT—SPM, were responsible for the differences among the twelve treated populations. The changes in the first two variables were responsible for the altered PA metabolism in the Col-0 and *phyA* populations without SPM treatment under L1 and L2 and, to a lesser extent, under L3 light treatment. The second group of three genes led to the discrimination of SPM-treated *phyA* mutant under L1 and L3, and the third group differentiated the SPM-treated Col-0 genotype at each of the lights and the mutant one under the L2 condition.

## 3. Discussion

PA biosynthesis is controlled by light (light quality and quantity) [38,39], and on the other hand, PAs are able to influence photosynthesis in several ways (reviewed in [24]). PhyA is a unique photoreceptor responsible for the high far-red light irradiance response of seedlings, and PhyA regulates various light-dependent responses at metabolite and gene expression levels; however, PhyA may regulate genes other than light-responsive ones through the interaction with corresponding transcription factors [23]. In addition, some results suggest the involvement of PhyA in mediating the blue light photoresponses, such as chloroplast biogenesis, phototropism, anthocyanin synthesis or stem elongation and shade-avoidance responses [16,17,18,19]. Although some limited information suggests a relationship between PhyA and PA metabolism under white and far-red light conditions [12], until now, no information has been available on the involvement of PhyA in blue light-influenced regulation of the PA metabolism in plants. Therefore, the present study attempts to uncover the impact of far-red and blue light on PA production through the investigation of *phyA* mutant plants, as well as changed light spectra and supplementation by exogenous PA.

The *phyA* mutation did not influence the shoot weight under the different light conditions compared to the adequate wild-type controls. At the same time, the light quality has more influence on the shoot weight than the mutation, as L2 and L3 light treatments increased it in both genotypes compared to the control white light (L1) condition. Similarly to these results, the biomass of individual monogenic *phyA* or *phyB* mutant plants was the same as that of the wild type, suggesting that the action of either phyA or phyB is sufficient for normal biomass production [42]. *Arabidopsis* leaf area and petiole length also showed a correlation with blue light irradiance levels [43], and a decrease in the R/FR ratio has also been reported to significantly increase petiole elongation and leaf area expansion of the Col-0 line [44]. In addition, the biomass parameters of the *phyA* mutant were similar to that of the wild type, even under far-red light supplementation [42].

Although the effects of exogenous Pas have been tested in *Arabidopsis* mutants, especially in PA metabolism mutants [35,45], no information is about the effect of exogenous SPM on *phyA* mutants. Previously we also demonstrated that one-day treatment with 0.5 mM SPM was enough to induce changes in PA metabolism in *Arabidopsis* Col-0 and salicylic acid-related mutants [36]. In the present study, the SPM treatment did not influence remarkable changes in the shoot weight or chlorophyll-*a* induction parameters of the plants, indicating that the plants were under optimal growth conditions. In order the better understand the biological effect of PA metabolism and its relationship with the PhyA, PA metabolism was investigated at metabolite and gene expression levels.

The expression of *AtADC2* was lower in the mutant plants under L1 but the same under L2 and L3 light conditions; in addition, it was downregulated by L2 and L3 treatments in Col-0 plants. This indicates that the expression of *AtADC2* is dependent on far-red, as well as blue light signaling and that this signaling pathway goes through PhyA. SPM treatment increased *AtADC2* expression in both genotypes, suggesting that exogenous SPM induced de novo synthesis of PUT. As *AtADC2* expression increased, especially in the case of the mutant plants, with a higher degree under L1 and L3 light conditions, these results support that PhyA is involved in the regulation of PUT synthesis, but also another pathway regulated by far-red light is probably included, for example, PhyB might play a role in the process [5]. As it has been demonstrated, PUT and SPD contents only decreased in *phyB* mutants under far-red light in contrast to the white light conditions [14]. It was also reported that the stimulating effect of red light on the activities of ADC and S-adenosylmethionine decarboxylase enzymes could be reversed with the application of far-red light, implicating Phy again in the regulation of PA biosynthesis. While blue light-induced changes in PA synthesis enzyme activity could not be reversed by far-red light, thus the influence of PA synthesis via different signal transduction pathways suggests the involvement of a separate blue-light receptor [46,47]. Despite the well-recognized differences between the expression of *AtADC2* in the two SPM-treated genotypes, as well as under different light regimes, SPM treatment increased the PUT level similarly in both genotypes, which was independent of the light conditions, suggesting that other processes than the synthesis are involved.

The *AtSPDS2* transcription levels did not differ pronouncedly between the two genotypes under L1, L2 and L3 conditions indicating less influence of PhyA and, in turn, far-red light on the regulation of SPD synthesis than on PUT synthesis. In addition, the lowest *AtSPDS2* expression level was found in both genotypes under the L3 light treatment, which suggests the negative impact of blue light on this PA biosynthetic enzyme. Nevertheless, no remarkable changes were observed in the SPD levels after the treatments. SPM treatment decreased the expression of *AtSPDS2* in both genotypes; in addition, it slightly decreased SPD levels under L1 and L2 conditions.

The expression level of *AtSPMS* downregulated in *phyA* mutant compared to the wild type under L1 and L2 light treatments, while under L3 light conditions, it was initially the lowest. These results further support the hypothesis that PhyA has a role in the synthesis of PAs (in this case, that of SPM), and blue light suppresses it. However, SPM treatment induced the highest increment under L3 in Col-0; a slight increase in both genotypes was induced under L2, while under L1, the transcript level of *AtSPMS* increased only in the mutant. This is in contradiction with the response of SPM non-treated plants, which means that the high concentration of SPM could influence PhyA signaling in opposite feedback, at least under white light conditions. Moreover, *AtSPMS* upregulation under L3 conditions after SPM treatment indicates that the high SPM level stimulated the production of SPM biosynthetic enzyme by a blue light signal. Despite the modifications of the enzyme expression, the pattern of SPM changes did not follow the tendency of *AtSPMS* expression levels, as the SPM concentration was very similar in both genotypes under the three applied light treatments. Exogenous SPM increased the SPM level in Col-0 and phyA under L1 and L2 light conditions equally, suggesting that SPM was transported from the roots to the leaves. Moreover, lower SPM accumulation under higher far-red light irradiance may have resulted from the conversion of SPM, but this process was not driven by PhyA. The SPM accumulation in SPM-treated Col-0 was double that of the phyA mutant under the L3 condition, which is clear evidence that PhyA-induced accumulation of SPM under higher blue light irradiance and high SPM concentrations and that PhyA is influenced by blue light. This process was probably regulated at the level of PA degradation and SPM back-conversion and not by PA synthesis. However, modification in the PA uptake transport mechanisms cannot be excluded too.

The *AtCuAO1* expression was independent of the light condition or the *phyA* mutation. However, SPM induced *AtCuAO1* expression in the mutant genotypes under L1 and L3 light conditions. Therefore, the gene expression pattern of *AtCuAO1* was partly similar to that of *AtADC2* under L1 and L3, suggesting that the higher SPM concentration-induced de novo synthesis of PUT was compensated by its higher degradation in the mutant plants. In turn, no differences were observed in the actual PUT contents. The expression level of *AtCuAO3* did not show pronounced changes but was downregulated under L3 conditions in Col-0. It indicates that higher blue light irradiance diminished the degradation of PUT under the control of PhyA. The initially higher *AtCuAO3* expression in *phyA* mutant under the L3 regime might reflect the involvement of PhyA in this process, which is similar to the above-mentioned modifications in *AtCuAO1* expression under SPM treatment combined with L3 conditions. Slight increases in *AtCuAO3* expression after SPM application in both genotypes under L1 light and in Col-0 under L3 indicate that this process is dependent on the actual level of PAs. It is possible that PAs might affect the phosphorylation of light receptors, probably not only PhyA [48] and enhance their role in gene transcription [49], or that PAs directly stimulate the translation of PhyA and other genes [50].

Excess SPM in the nutrition solution increased SPM content in the leaves, which in turn induced the expression of *AtPAO2* under all the light conditions regardless of the genotypes. At the same time, *AtPAO5* levels were initially the lowest under L3 conditions in Col-0 plants indicating suppressed back-conversion and suggesting again the influence of PhyA under intensive blue light irradiance (similarly to genes of biosynthetic: *AtADC2*, *AtSPDS* and *AtSPMS*, and degradation enzymes: *AtCuAO3*). SPM treatment mostly induced the *AtPAO5* expression, especially under L2 conditions, but under L3 conditions only in Col-0 plants. Under the L2 condition in both genotypes, the initial level of *AtPAO5* expression was higher than that was detected under L1 conditions in Col-0 or *phyA* mutant plants. This higher expression already without SPM treatment under far-red light suggests a higher inducibility of the back-conversion mechanism, which in turn can be responsible for the lower SPM accumulation after SPM treatment under L2 light condition. The initially higher transcript level of *AtPAO5* in the mutant plant compared to the wild type under L3 conditions can be again in relation to the observed lower SPM content in SPM-treated mutants under L3 light conditions.

A close, positive relation between PUT the SPM content, and both PUT and SPM contents with *ADC2*, *SPMS* and *PAO2* expression levels showed that the increase in SPM content not only resulted from the uptake of SPM from the hydroponic solution but the in vivo synthesis of SPM was also induced. SPM treatment also increased the PUT content due to the activation of the in vivo PUT synthesis, and parallel with it, the more intensive SPM-SPD-PUT back-conversion pathway could be responsible, too. Close, negative relations between PUT or SPM levels and SPD content and *SPDS2* transcript level indicate that the accumulation of PUT and SPM occurred at the expense of SPD content. Furthermore, a strong, negative correlation between SPD content and *PAO2* and *PAO5* expression levels confirmed that lower SPD content has resulted from not only the decreased *SPDS* expression but also the induced back conversion. The PCA analysis reflected that L3 light conditions had a specific effect on the PA metabolism, and SPM treatment was a more significant factor for the distinction of treated plants. Without SPM treatment, the treatments groups of L1 and L2 light conditions, both of the wild type and mutant genotypes, were separated from that of the L3 light, while after SPM treatment, the treatment group of mutant plants under L1 and L3 were similar to each other and partly different from the others (mutant under L2 light and wild type under all light conditions).

Previously, we have demonstrated that among PAs applied hydroponically, especially SPM-induced rapid PA responses in the leaves of Col-0 *Arabidopsis* plants under white light conditions. The SPM application induced the PUT accumulation due to the increased expression level of *AtADC2*; the level of SPD did not change; however, the transcript levels of *AtSPDS1* and *two* slightly decreased, while the expression of *AtSPMS* increased [36]. These results indicate that SPM could influence the de novo synthesis of PAs. Partly similar results were found in the present experiment, as SPM induced the synthesis of PUT and activated the expression of *AtADC2* and *AtSPMS* but decreased that of the *AtSPDS2*. In wheat, it was also demonstrated that the effect of supplementary blue and far-red light was opposite on the PA metabolism-related gene expression in the leaves; in addition, PA treatment could partly reverse these differences [39]. Nevertheless, in the present study, we first demonstrated that the responses of wild-type and *phyA* mutant plants are partly different and depend on the light spectral conditions. Our results on changes induced by SPM treatment reflected that PhyA under blue light might primarily inhibit SPM back-conversion and subsequently downregulate PUT synthesis.

## 4. Materials and Methods

### 4.1. Plant Material, Plant Growth Conditions and Treatments

In the present experiment, the *phyA*-T mutant *Arabidopsis* (SALK_014575C), T-DNA insertion line in Col-0 background [51] was investigated, where Col-0 ecotype was used as control. Seeds were obtained from the European Arabidopsis Stock Centre (NASC, Sutton Bonington Campus, Loughborough, LE125RD, United Kingdom). The plants were self-pollinated for two generations, and the presence of the mutation was confirmed by genotyping (Supplementary Figure S2). The *phyA* mutant is a T-DNA insertion line created by SALK Institute/SAIL, so the genotyping primers (Supplementary Table S1) were designed with the help of signal.salk.edu/tdnaprimers.2.html website.

Plants were cultivated hydroponically using an Araponics system (Araponics, Liège, Belgium). For hydroponic solution, 25% Murashige and Skoog Medium (Duchefa) were used. Plants were grown in a Conviron GB-48 phytochamber (Controlled Environments, Winnipeg, MB, Canada) under control conditions at 22/20 °C with 8/16 h light/dark period and 75% humidity for 28 days.

Plants were grown under different spectral conditions at the same light intensity from germination (100 µmol m^−2^ s^−1^). Three different light regimens were established using modules equipped with a continuous wide spectrum LED (Philips Lumileds, LXZ2-5790-y) and three narrow bands of LEDs with the dominant wavelengths of 448 nm (Philips Lumileds, LXZ1-PR01); 655 nm (Philips Lumileds, LXZ1-PA01); 750 nm (Edison Edixeon, 2ER101FX00000001). All light source modules were equipped with these LEDs, and each type of LED could be independently controlled within the module. The spectral composition of the three applied light treatments used in the experiments is described in Table 2. The spectral composition was chosen with some modifications based on our previous study, where the effects of supplementary blue and far-red light and their combination was investigated on PA metabolism, and blue light caused a drastic decrease in the gene expression level of PA metabolism-related gene expression, while the far-red light-induced slight increase of them in the leaves of wheat plants [39].

Four-week-old plants were treated with 0.5 mM spermine (SPM) for one day, which was added directly to the nutrient solution. The concentration and the duration of SPM treatment were chosen based on our previous study, where 1 day 0.5 mM SPM treatment was sufficient and induced the most pronounced changes in polyamine metabolism compared to putrescine or spermidine application [36]. Thereafter shoots were collected, measured for shoot weight parameter, and fully developed leaves were frozen immediately in liquid nitrogen. Samples were stored at −80 °C until further analysis.

### 4.2. Chlorophyll-A Fluorescence Induction (FI) Analysis

The FI analysis was carried out using pulse amplitude modulated fluorometer (PAM) with a blue LED-Array Illumination Unit IMAG-MAX/L (λ = 450 nm) (Imaging-PAM M-Series, Walz, Effeltrich, Germany) on the fully expanded leaves of Arabidopsis plants, which were exposed to dark for 15 min in order to reach the open state of the acceptor side of the electron transport chain. The determination of the maximum quantum efficiency (Fv/Fm) and the actual quantum yield [Y(II)] of photosystem 2 was carried out as it was described in [39].

### 4.3. Polyamine Analysis

Leaf samples were homogenized in 2 mL 0.2 N HClO_4_, and the homogenates were centrifuged at 4 °C for 10 min, with 10,000× *g*. The supernatant was used for the pre-column derivatization with dansyl chloride. PUT, SPD, SPM, and one of the products of terminal catabolism of SPD and SPM, 1,3-diaminopropane (DAP), were analyzed on a reverse phase Kinetex column (C18, 100 × 2.1 mm, 5 μm, Phenomenex, Inc., Torrance, CA, USA) by HPLC consists of a W2690 separation module and a W474 scanning fluorescence detector with excitation at 340 nm and emission at 515 nm (Waters, Milford, MA, USA) according to [39]. 2 μL of the derivatized sample injected onto the column, two types of solvents were used during the measurement (A: 44%ACN C: ACN: MeOH = 7:3). Gradient program was used for separation and during the analysis the flow rate was 0.5 mL min^−1^, and the column temperature was 40 °C. Data evaluation was performed using the Millenium32 program (WATERS, Milford, MA, USA).

### 4.4. Gene Expression Analysis

For gene expression studies, fully developed leaves of the wild type and the phyA mutant were taken at the end of the treatments and immediately stored in liquid nitrogen. Total RNA extraction and cDNA synthesis was performed as described in [36] using TRI Reagent, Direct-zol™ RNA MiniPrep Kit (Zymo Research, Irvine, CA, USA), including on-column Dnase I treatment for RNA purification and with M-MLV Reverse Transcriptase (Promega Corporation, Madison, WI, USA) and oligo (dT)18 (Thermo Fisher Scientific Inc., Wilmington, MA, USA) for cDNA synthesis according to the manufacturer’s instructions. For RT-qPCR measurements, a BioRad CFX96 Touch Real-Time Detection System was used with 1 µL 4-fold diluted cDNA, 200 nM forward and reverse primers (primer sequences are available in Table 3), 2.5 uL PCRBIO Mastermix (PCR Biosystem Ltd., London, United Kingdom) and 2.5 ul molecular grade water. Relative transcript levels were determined with the 2^−ΔΔCt^ method [52], with AtActin8 as the internal control gene, and values were compared to the control Col-0 genotype grown under L1 light condition.

### 4.5. Statistical Analysis

The results are the means of 14 biological replicates for biomass parameters, five biological replicates for chlorophyll-a fluorescence induction measurement, and at least three biological replicates for chromatographic determinations. All reactions for gene expression analyses were performed in triplicate using 3 biological and 3 technical repetitions. The data were statistically evaluated using the standard deviation in Microsoft Excel (STDEV.S function) with n ≥ 3. Different letters indicate statistically significant differences (*p* < 0.05) between multiple groups (one-way ANOVA with Duncan’s post hoc test was performed using SPSS 16.0). Pearson’s correlation coefficients were calculated using the SPSS 16.0 version. The principal component analysis (PCA) was carried out in the R environment (ver. 4.0.3) using the packages FactoMineR, factoextra and ggplot2.

## 5. Conclusions

Light spectrum significantly affects PA metabolism-related genes. Under the used experimental conditions, the blue light influences the synthesis side, while the far-red light affects on the catabolism and back-conversion of the PAs. The observed responses to elevated far-red light were found to be less dependent on PhyA, as the mutation alone induced no remarkable differences. Contrarily, the blue light response seems to be highly dependent on PhyA signaling. PhyA under higher blue light irradiance downregulated the transcript levels of genes involved in PA synthesis, back-conversion and degradation compared to the white light.

**Figure 6:**
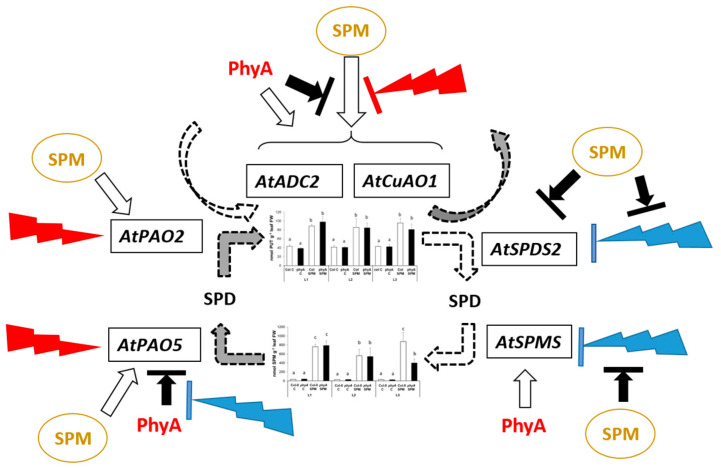
Schematic mode of action of spermine (SPM) treatment on polyamine metabolism under different light spectral compositions and influence of PhyA on it. PUT: putrescine, SPD: spermidine, SPM: spermine content, *AtADC2*: arginine decarboxylase 2, *AtSPDS2*: spermidine synthase 2, *AtSPMS*: spermine synthase, *AtCuAO3*: cooper amine-oxidase 3, *AtPAO2* and *AtPAO5*: polyamine oxidase 2 and 5. Blue lightning arrows indicate blue light treatment, red lightning arrows indicate far-red light treatment, SPM indicates spermine treatment, and PhyA means the presence of the wild type of phytochrome A. Different letters indicate statistically significant differences at *p* < 0.05 level, using Duncan’s post hoc test.

Our results demonstrated that *phyA* mutation has some influence on PA metabolism. The PA metabolism-related gene expression of the wild type and the *phyA* mutant plants responded more similarly under white and far-red light conditions than under blue light conditions. The highest differences between the two genotypes were observed under the blue light treatment. Nevertheless, the PA contents were similar under all the light conditions in the two genotypes without SPM application, suggesting that a stable PA pool is important for normal plant growth conditions even under different light spectral conditions. The proper shift in PA levels required a well-maintained dynamic balance of PAs through fine-tuning of PA metabolism. However, SPM treatment brought out more differences in metabolite and gene expression levels. After SPM treatment, the blue dominant spectral regime has more similar effects on PUT synthesis/catabolism and SPM to PUT canalization to the white light than the far-red light conditions. The additive effect of differences observed on the synthesis, back-conversion and catabolism side of PUT metabolism may be responsible for the similar PUT content pattern under all light conditions, even in the presence of an excess SPM. However, the regulatory system was well fine-tuned, and no alterations were detected at the level of PAs; only the SPM content differed significantly between the two genotypes after SPM treatment under blue light conditions. Figure 6. presents the hypothesized mode of action of SPM treatment on PA metabolism under different light spectral compositions and the influence of PhyA on it.

Nevertheless, as PhyA may not only serve as a photoreceptor but regulates genes other than light-responsive ones (including members of morphogenesis, hormone, stress, and defense signaling pathways) through the interaction with corresponding transcription factors, further research is necessary to uncover the proper mechanisms of interaction between PAs and light-regulated processes.

## Figures and Tables

**Table 1:** Correlation analysis on the polyamine metabolism-related parameters. Strong significant correlations at 0.05 are in bold; a positive correlation is highlighted in green, and a negative correlation is highlighted in red. PUT: putrescine content, SPD: spermidine content, SPM: spermine content, *ADC2*: expression level of arginine decarboxylase 2, *SPDS2*: expression level of spermidine synthase 2, *SPMS*: expression level of spermine synthase, *CuAO1* and *CuAO3*: expression level of cooper amine-oxidase 1 and 3, *PAO2* and *PAO5*: polyamine oxidase 2 and 5 genes.

	PUT	SPD	SPM	*ADC2*	*SPDS2*	*SPMS*	*CuAO1*	*CuAO3*	*PAO2*	*PAO5*
**PUT**	1									
**SPD**	**−0.555**	1								
**SPM**	**0.915**	**−0.572**	1							
***ADC2***	**0.688**	**−0.379**	**0.617**	1						
***SPDS2***	**−0.524**	**0.559**	**−0.502**	−0.188	1					
***SPMS***	**0.737**	**−0.293**	**0.753**	**0.710**	−0.139	1				
***CuAO1***	**0.414**	−0.147	**0.345**	**0.820**	−0.095	**0.553**	1			
***CuAO3***	0.169	−0.124	0.159	**0.413**	0.130	**0.261**	**0.410**	1		
***PAO2***	**0.834**	**−0.593**	**0.829**	**0.688**	**−0.431**	**0.666**	**0.470**	**0.399**	1	
***PAO5***	**0.469**	**−0.500**	**0.423**	0.157	−0.135	**0.334**	−0.050	**0.260**	**0.423**	1

**Table 2:** Characteristics of the applied three light regimes. Light treatments are colored according to their typical characteristic; the light source for (L1: white color) was a continuous wide-spectrum LED; in the case of the L2 regimen (red color) elevated far-red component was applied, while under L3 light condition (blue color) the blue light component was the most dominant.

Treatment	Intensity PAR (µmol m^−2^ s^−1^)	Blue µW/cm^2^ (400–500 nm)	Green µW/cm^2^ (500–600 nm)	Red µW/cm^2^ (600–700 nm)	Far-red µW/cm^2^ (700–800 nm)	Blue/ Red	Red/ Far-Red	Blue%	Green%	Red%	Far-Red%
L1	100	400	640	1020	30	0.39	34	19.14	30.62	48.8	1.44
L2	100	450	680	930	240	0.48	3.88	19.57	29.57	40.43	10.43
L3	100	890	650	690	30	1.29	23	39.38	28.76	30.53	1.33

**Table 3:** Primer sequences.

Gene Name	Primer Sequences (5′ → 3′)		Amplicon Size (bp)	References
*AtActin8*	forward	TTACCCGACGGACAAGTGATC	73	[53]
	reverse	ATGATGGCTGGAAAAGGACTTC		
*AtADC2*	forward	GCGATGGACCACACAGCTTT	64	
	reverse	AGAACATCCGCTGAGGACTGA		
*AtSPDS2*	forward	TTGCCCGTGAAGAGACCTAGA	72	
	reverse	TCCACCGTTCTCTGTTTCCAT		
*AtSPMS*	forward	TGGCTCCATACTCATCTTATTGAA	72	
	reverse	CGCATAGTGAACACTTTTGAATG		
*AtPAO2*	forward	GGAATGCCGGAAGATCTTCCGTGATTGTGATCGG	142	[54]
	reverse	CGATTCCAACACCGAGATTTGCATACTCCATGCAGC		
*AtPAO5*	forward	GTTGGGATGAACCAGAAGGA	132	[55]
	reverse	GAGGAGCCTCGGTAAGAAGA		
*AtCuAO1*	forward	AGCTGGCGACATTCTGAGAT	238	[29]
	reverse	GTCCAGCATCATCCTCCCTA		
*AtCuAO3*	forward	GTAAGTTTGTGCCACTCCCCC	153	
	reverse	GCCACTCGACAAAGTACCCCC		

## Data Availability

Data is contained within the article or Supplementary Material.

## Supplementary Materials

The following supporting information can be downloaded at: https://www.mdpi.com/article/10.3390/plants12081689/s1, Supplementary Figure S1. (a) The actual quantum efficiency of photosystem II [Y(II)] chlorophyll-a fluorescence induction parameter determined at the steady state level of photosynthesis of the leaves (b) of wild type (Col-0) and mutant (*phyA*) Arabidopsis plants grown under three different light regimes (L1: blue%: 19.139, green%: 30.62, red%: 48.8 and far-red%: 1.44; L2: blue%: 19.57, green%: 29.57, red%: 40.43 and far-red%: 10.43; L3: blue%: 39.38, green%: 28.76, red%: 30.53 and far-red%: 1.33) treated with or without 0.5 mM spermine (control: C and spermine: SPM). Values are means ± SD (n = 5 for Y(II)). Different letters indicate statistically significant differences at *p* < 0.05 level, using Duncan’s post hoc test. (b) The chlorophyll fluorescence imaging screens of Y(II) in the leaves under different treatments and light conditions created by the Imaging PAM instrument. The colored bar shows the range of pixel intensity values. Supplementary Figure S2. Confirmation of T-DNA inzertion in *phyA* mutant. PCR of the genomic DNA of Col-0 and phyA mutant plants. PCR fragments were obtained using the primer pairs LP and RP and/or LBb1.3 (T-DNA specific primer) and RP, respectively. Primers are listed in Supplemental Table S1. Supplementary Table S1. Primer sequences for genotyping of *phyA* mutation.
