# Supplementary material for: Influence of a phyA Mutation on Polyamine Metabolism in Arabidopsis Depends on Light Spectral Conditions
Source: Plants (Basel). 2023 Apr 18;12(8):1689. doi: 10.3390/plants12081689 (PMC10146636; doi:10.3390/plants12081689)
Supplement: Supplementary file 1 [file plants-12-01689-s001.zip › plants-2324873-supplementary.pdf]

Suppl. Figure S1. (a) The actual quantum efficiency of photosystem II [Y(II)] chlorophyll-*a* fluorescence induction parameter determined at the steady state level of photosynthesis of the leaves (b) of wild type (Col-0) and mutant (*phyA*) *Arabidopsis* plants grown under three different light regimes (L1: blue%: 19.139, green%: 30.62, red%: 48.8 and far-red%: 1.44; L2: blue%: 19.57, green%: 29.57, red%: 40.43 and far-red%: 10.43; L3: blue%: 39.38, green%: 28.76, red%: 30.53 and far-red%: 1.33) treated with or without 0.5 mM spermine (control: C and spermine: SPM). Values are means ± SD (n=for Y(II)). Different letters indicate statistically significant differences at $p < 0.05$ level, using Duncan's post hoc test. (b) The chlorophyll fluorescence imaging screens of Y(II) in the leaves under different treatments and light conditions created by Imaging PAM instrument. Coloured bar shows the range of pixel intensity values.

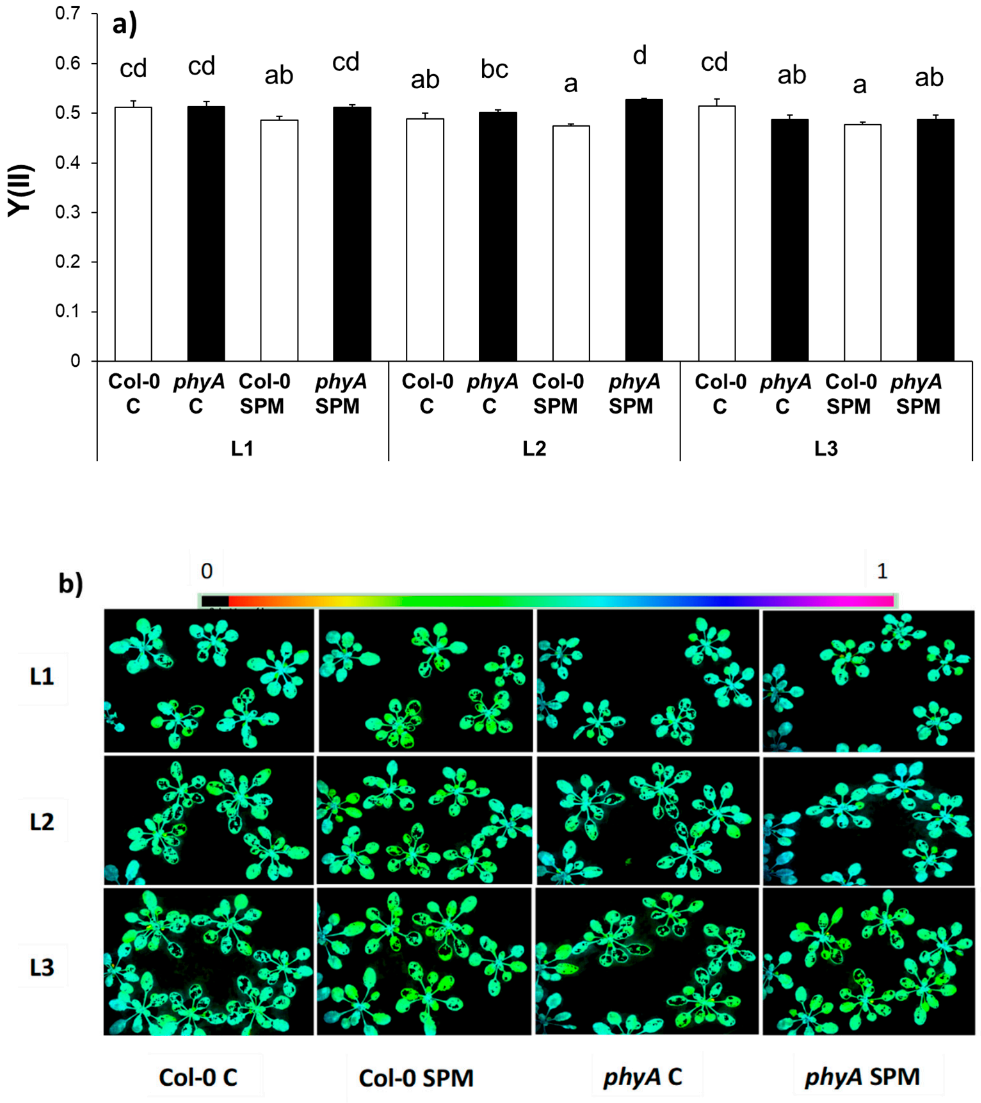

Suppl. Figure S2. Confirmation of T-DNA inzertion in *phyA* mutant. PCR of the genomic DNA of Col-0 and *phyA* mutant plants. PCR fragments were obtained using the primer pairs, LP and RP and/or LBb1.3 (T-DNA specific primer) and RP, respectively. Primers are listed in Supplemental Table 1.

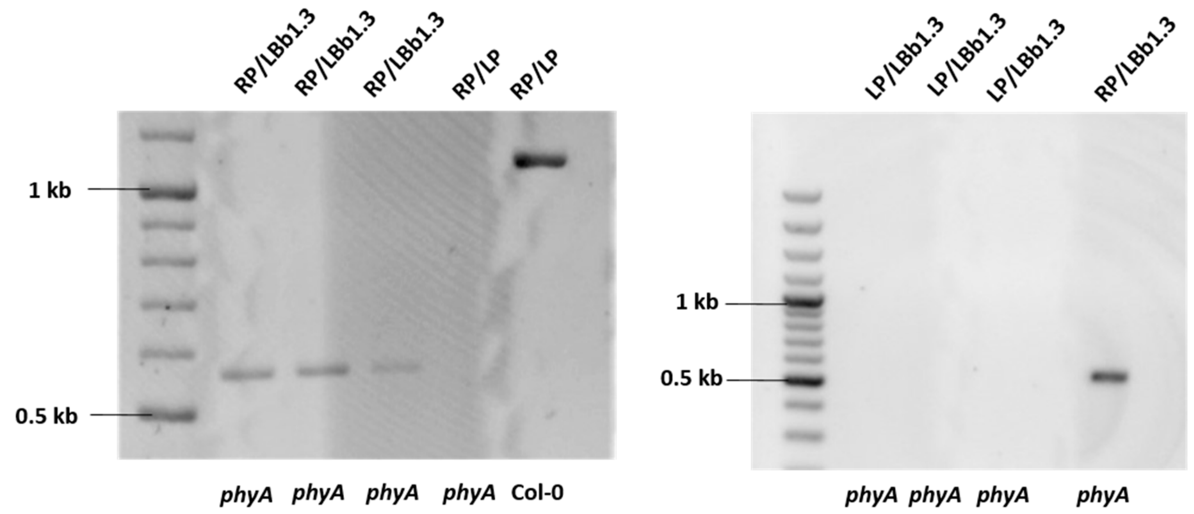

Suppl. Table S1. Primer sequences for genotyping of *phyA* mutation.

Gene name	Primer sequences (5' → 3')	
phyA mutant (NASC code: 66049)	phyA_LP	CCAGTCAGCTCAGCAATTTTC
	phyA_RP	AATGCAAAACATGCTAGGGTG
	phyA_LBb1.3	ATTTTGCCGATTTCGGAAC
